# Drought-Tolerance QTLs Associated with Grain Yield and Related Traits in Spring Bread Wheat

**DOI:** 10.3390/plants11070986

**Published:** 2022-04-04

**Authors:** Sahar Bennani, Ahmed Birouk, Mohammed Jlibene, Miguel Sanchez-Garcia, Nasserelhaq Nsarellah, Fatima Gaboun, Wuletaw Tadesse

**Affiliations:** 1 Plant Breeding and Conservation of Phytogenetic Genetic Resources Department, National Institute of Agricultural Research, Rabat 10101, Morocco; fatima.gaboun@inra.ma; 2 Department of Production, Protection and Biotechnology of Plants, Agronomy and Veterinary Hassan II Institute, Rabat 10101, Morocco; a.birouk@iav.ac.ma; 3 National Federation of Milling, Casablanca 20000, Morocco; jlibene.mohammed@gmail.com (M.J.); nsarellah@yahoo.com (N.N.); 4 Biodiversity and Crop Improvement Program, International Center for Agricultural Research in the Dry Areas, Rabat 10101, Morocco; m.Sanchez-Garcia@cgiar.org (M.S.-G.); w.Tadesse@cgiar.org (W.T.)

**Keywords:** *Triticum aestivum* L., drought tolerance, selection criteria, quantitative trait loci, GWAS

## Abstract

The present research aims to identify the efficient combination of drought-tolerance selection criteria and associated quantitative trait loci. A panel of 197 bread wheat genotypes was evaluated for yield- and drought-tolerance-related traits in two environments (favorable and semiarid) for 2 years (2015–2016). Grain number, biomass, number of fertile spikes per plant and ground cover exhibited a significant correlation with grain yield and constitute potential secondary selection criteria for yield under drought conditions. About 73 significant marker–trait associations were detected along various chromosomal positions. The markers “*wsnp_Ex_Rep_c67786_66472676*” and “*ExcalibuR_c24593_1217*” exhibited important genetic gains associated with yield increase under drought (11 and 7%, respectively). The markers “*KukRi_c94792_127*” and “*wsnp_Ex_c298_580660*” showed a significant correlation with grain yield, biomass and grain number and were associated with a significant increase in yield performance at the semiarid site (+6 and +7%, respectively). The ground cover was found associated with grain yield and biomass through the markers “*wsnp_Ex_Rep_c67786_66472676*” (+11%) and “*KukRi_c49927_151*” (+10%). One marker “*TduRuM_contig25432_1377*” on chromosome 5B at 20 cM was consistently correlated with the number of fertile spikes across both environments. Further research should be considered to validate the efficiency of these markers to undertake selection for drought tolerance under various environments and genetic backgrounds.

## 1. Introduction

Bread wheat (*Triticum aestivum* L.) is one of the most important staple crops in the world. It represents an important source of calories and proteins in most developing countries. Drought remains one of the main stresses threatening wheat production, especially under climate change conditions [1,2,3]. From this perspective, the release of high yielding and drought resilient varieties may help to improve wheat productivity and stability under climate change conditions [4,5,6]. However, breeding for drought tolerance is generally hampered by the high variability of drought scenarios in line with the genetic complexity of drought tolerance and associated plant response mechanisms. Grain yield has low heritability (<20%) over variable stress intensities and unpredictable environmental conditions [7,8,9], which necessitates the identification of reliable secondary indirect selection criteria to improve the efficiency of selection for drought tolerance [10]. Yield components and some morphological and physiological traits have been considered as relevant traits to screen germplasm for drought tolerance since these traits have usually shown significant correlation with grain yield. Grain number, number of productive spikes, biomass and thousand kernel weight [11], plant height [11,12], early ground cover [13], stomatal conductance [14], chlorophyll content [15], carbon isotope discrimination [16], and canopy temperature [17] are some of the traits reported to have significant correlations with grain yield.

For millennia, plant breeding has played a key role in developing high yielding and stress tolerant varieties using conventional technologies. During the last century, a wide range of molecular marker technologies and big data association software have been developed to assist breeding programs to study and separate the confounding effects of the environment during selection [11].

Genome wide association studies (GWAS) have been an effective and powerful tool in identifying candidate genes and mapping complex quantitative traits for many species. Taking advantage of the available large populations and high throughput sequencing technology, association mapping has gained importance over linkage mapping for the identification and mapping of marker trait associations (MTAs) without the need to develop a bi-parental mapping population [18,19,20]. To date, several association mapping studies were carried out in wheat for drought tolerance [11,19,21,22]. Initially, SSR markers were widely used, and stress-related QTLs have been reported [23,24]. Recently, advances in DNA sequencing have enabled the development of more suitable markers for the dissection of complex traits on complex genomes [11,20,25,26]. Through next-generation sequencing, several high-density automated genotyping platforms have been developed for staple crops, enabling a better coverage of the genome with high quality markers and cost effectiveness per data point [27,28,29]. Several consensus maps in wheat have been developed, such as 9k, 35k, 90k and 660k SNPs arrays [30,31,32,33]. From these arrays, wheat 50k and 15k SNP platforms are now available for selecting important traits in wheat breeding programs [20].

In the current study, a large panel of 197 diverse bread wheat lines from ICARDA germplasm is phenotyped and sequenced using 15k SNP markers derived from the 90k Illumina iSelect arrays [31]. Our hypothesis is that the large diversity of both the genetic panel and SNP markers could reveal interesting associations with drought-related traits under Moroccan environments. This study aims to (i) identify the best combination of secondary traits for drought tolerance screening, (ii) detect relevant MTAs related to these traits and to (iii) select the best high yielding and drought-tolerant genotypes.

## 2. Results

Both locations experienced periods of drought, especially in 2016 season. The humid site “Taoujdate” showed higher average yield with 3.87 t ha^−1^ (3.8 and 4.1 ha^−1^ in 2015 and 2016 seasons, respectively) compared to 3.02 t ha^−1^ (3.3 and 2.8 ha^−1^ in 2015 and 2016 seasons, respectively) for the semi-arid site “Sidi El Aidi”. ANOVA showed highly significant effects of genotypes for all traits at both stations. Similarly, significant genotypes x year interactions were observed, especially at the Sidi El Aidi experimental site.

### 2.1. Pearson Correlation Analysis

Grain yield expressed significant positive correlation with biomass (BM), grain number (GN) (r > 0.83; *p* < 0.001), and number of fertile spikes per plant (NFSP) for both experimental sites. The ground cover (GC) showed high positive correlation with grain yield at the semi-arid station (r = 0.75; *p* < 0.001), while it was very weak (r = 0.16; *p* = 0.026) at the optimum conditions. There was no significant correlation observed between days to flowering (DTF) and grain yield (Table 1).

### 2.2. Regression Analysis

The regression model was performed to determine the weight of each trait on yield performance. GN and BM constitute the best predictors, explaining a range from 70 to 80% of the yield variation. Furthermore, NFSP and GC explained a significant part of yield variation, especially at the dry environment (47 and 56%, respectively for NFSP and GC) (Table 2).

Path analysis showed that GN had the highest direct effect on both environments (0.61 and 0.55, respectively at Sidi El Aidi and Taoujdate). Under the dry environment, the GC also had a significant direct effect on grain yield (0.31), whereas NFSP and BM act indirectly supporting GN effect. On the other hand, only BM had an important indirect effect through GN at Taoujdate conditions (0.58) (Table 3).

Additionally, GC was also able to explain a great percentage of BM, GN and NFSP variations (79, 80 and 47%, respectively) at the Sidi El Aidi station as compared to the low variations under Taoujdate conditions (Table 4).

### 2.3. Genetic Analysis

#### 2.3.1. Marker–Trait Associations

A total of 73 significant marker–trait associations (MTAs) was detected along various chromosomes for yield- and drought-related traits at *p* < 0.001 (Supplemental Tables S1 and S2). Most of these MTAs were located on the B genome (13 MTAs for the Taoujdate and 28 MTAs for the Sidi El Aidi sites), and the A genome (9 and 7 MTAs, respectively, for Taoujdate and Sidi El Aidi sites). The least number of associations (four and two MTAs for Taoujdate and Sidi El Aidi sites, respectively) were located on the D genome. The highest number of MTAs was located on chromosomes 2B (26), followed by nine MTAs on chromosome 5B, four MTAs on chromosomes 3A and 5A, and three MTAs on chromosomes 1A, 6A and 7B.

**Figure 1:**
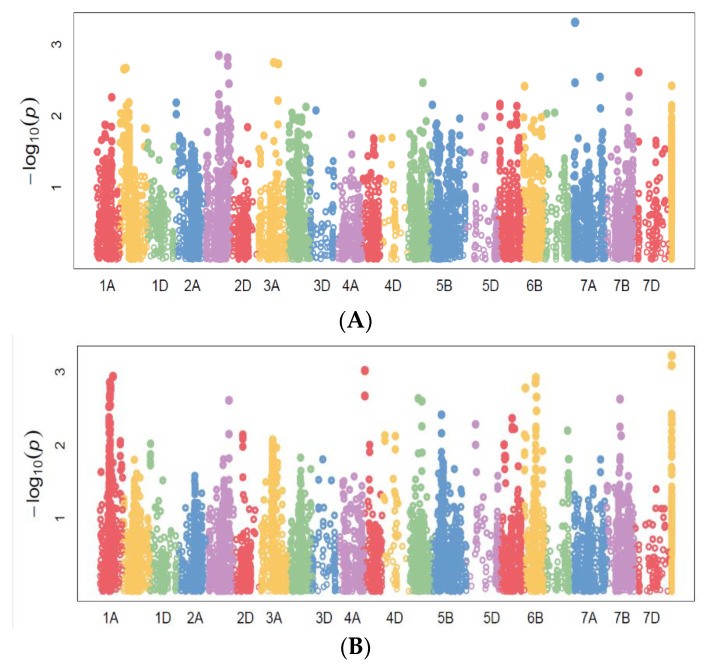
Manhattan plots showing SNP markers associated with grain yield at (**A**) Sidi El Aidi and (**B**) Taoujdate.

**Figure 2:**
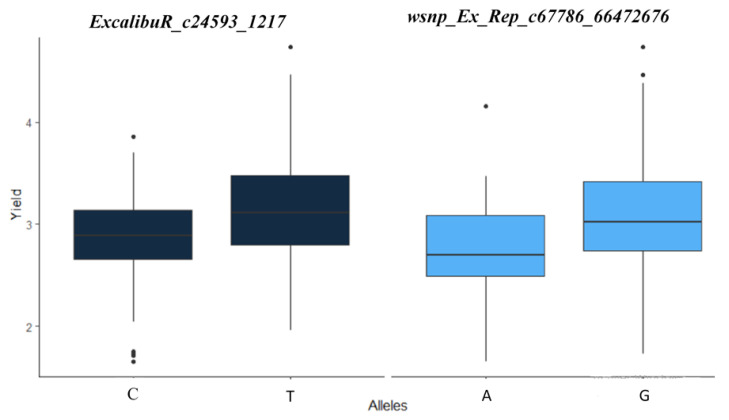
Average grain yield (t ha^−1^) depending on the base pair of single nucleotide polymorphism of the two markers “ExcalibuR_c24593_1217” (7A, 42 cM) and “*wsnp_Ex_Rep_c67786_66472676*” (3A, 110 cM) at the Sidi El Aidi station.

For grain yield (GY), three highly significant markers were located on chromosomes 4B (*BS00067775_51*) at the favorable rainfed station “Taoujdate” with significant increase in yield (+0.26 t/ha, +6.5%), in addition to the markers “*TduRuM_contig14482_1013*” and “*RAC875_c78248_154*” (Figure 1). At Sidi El Aidi, the lines carrying the T base from the marker “*ExcalibuR_c24593_1217*” (7A, 42cM) showed a significant yield increase of 10% (+0.32 t/ha) under drought conditions. Moreover, the lines carrying the G base at the marker “*wsnp_Ex_Rep_c67786_66472676*” (3A, 110 cM) increased their yield by 11% (3.08 t/ha; +0.38 t/ha) at the dry site. All these markers accounted for 5 to 7% of the variance (Figure 2).

Regarding yield components, six MTAs were associated with BM on chromosomes 1A, 5D and 3A at the Taoujdate station, while six significant associations were identified at the Sidi El Aidi site on chromosomes 1D, 2B and 2D, at *p* < 0.001. One association was common on chromosome 1A at 38 cM, related to the marker “*TduRuM_contig42479_3800*” for the semi-arid site and the marker “*tplb0043h23_1346*” for the favorable rainfed environment. The GN had one significant association at *p* < 0.001 related to the marker “*CAP12_c3807_144*” at the Taoujdate station, while seven MTAs were detected at Sidi El Aidi on chromosomes 1A, 1D, 2B, 2D, 3A, 5B and 7B (*p* < 0.003). The marker “*RAC875_c34888_65*” (1A, 35 cM) identified at Taoujdate is only 3 cM far located from the marker “*TduRuM_contig42479_3800*” (1A, 38 cM) at the Sidi El Aidi site. Twelve MTAs were detected for NFSP on chromosomes 5B, 7B, 7D and 2B for Taoujdate at *p* < 0.001, whereas seven MTAs were associated with the chromosomes 5B and 7B at Sidi El Aidi at *p* < 0.003. One marker on chromosome 5B at 20 cM (*TduRuM_contig25432_1377*) was consistent across both the Taoujdate and Sidi El Aidi locations.

Finally, TKW recorded the lowest number of MTAs. One MTA was detected on chromosome 3B at Taoujdate, while one MTA was linked to the chromosome 1B (96 cM) at the Sidi El Aidi site. The highest number of significant MTAs were detected for GC on the chromosomes 7B, 1D, 5A, 7A for Taoujdate and on the chromosomes 2B, 3A, 6A and 6B for Sidi El Aidi at *p* < 0.001 (Supplemental Figure S1).

#### 2.3.2. Common Markers

**Figure 3:**
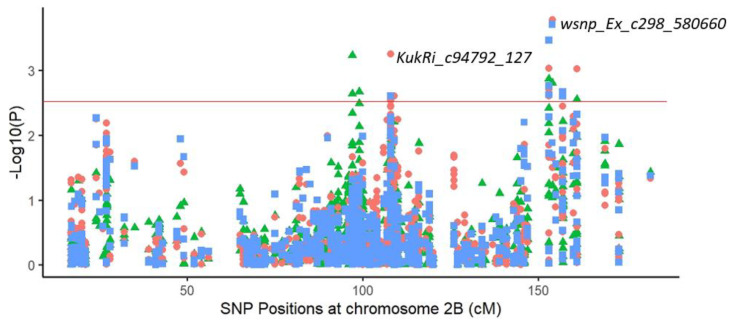
Genome-wide association study results using 940 single nucleotide polymorphism (SNP) markers on chromosome 2B in a 197 spring bread wheat population, showing pleiotropic effects for grain yield (triangle), biomass (dots) and grain number (square) in the Sidi El Aidi station. The horizontal line shows the −log10 (*p*) = 2.5 threshold.

Many common chromosomes positions and/or markers were found among the studied traits at the dry site. Regarding makers and chromosome positions implying grain yield and other traits, a common marker “*KukRi_c94792_127*” was linked to GY, BM and GN on chromosome 2B (153 cM). This marker allowed a yield increase of 7% (+0.22 t/ha) for the genotypes holding the “G” base in comparison with the “A” base. At the same chromosome, the marker “*wsnp_Ex_c298_580660*” (154 cM) was detected for the same traits and exhibited a significant yield increase of 11% (Figure 3).

**Figure 4:**
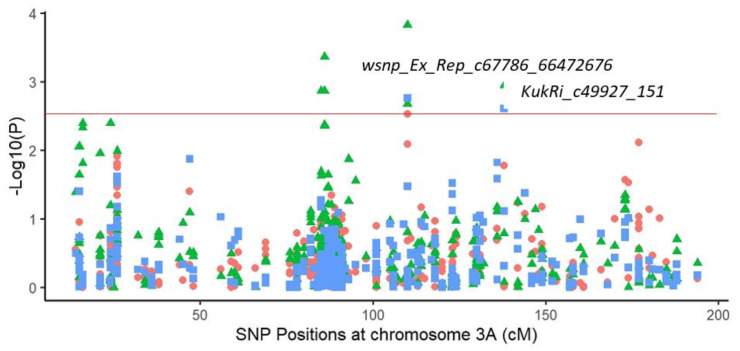
Genome-wide association study results using 425 single nucleotide polymorphism (SNP) markers on chromosome 3A in a 197 spring bread wheat population, showing pleiotropic effects for grain yield (square), biomass (dots) and ground cover (triangle) in the Sidi El Aidi station. The horizontal line shows the −log10 (*p*) = 2.5 threshold.

On the chromosome 3A, GC was linked to GY and BM at 110 cM with the “*wsnp_Ex_Rep_c67786_66472676*” marker. The A allele has a negative effect on GY and a positive effect on GC and BM. GY was also associated with GC at 138 cM with the marker “*KukRi_c49927_151*”, leading to a yield improvement of 0.37 t/ha (+10%) when holding the T allele (Figure 4).

Grain yield was associated with GN through the marker “*ExcalibuR_c24593_1217*” on the chromosome 7A at 42 cM. The effect of the allele C was positive on all traits. The GN and BM have also several common markers on chromosome 1A, 2B, 2D, and 7B (Supplemental Table S1).

At the “Taoujdate” site, grain yield and GC shared the same position on chromosome 6B. The grain number trait shared common markers on chromosomes 1A, 1B and 5B with BM, and on chromosome 7B with NFSP. The GC and TKW shared also a common position on chromosome 5A. GY was associated with NFSP and TKW at the same position on chromosome 5A (Supplemental Table S2).

### 2.4. Best Performing Genotypes

The derivative synthetic lines “OPATA/RAYON//KAUZ/3/2 * MILAN/DUCULA” and “PFAU/MILAN” achieved the highest yield performances under stressed and optimum conditions. Their grain yield varied from 4.5 to 4.7 t/ha under drought conditions and from 4.7 to 4.5 t/ha under a favorable environment with a respective genetic gain of 35 and 26%. These lines incorporated five to six of the main identified markers, namely “*wsnp_BG263521B_Ta_2_1*” (T), “*ExcalibuR_c24593_1217*” (T), “*wsnp_Ex_Rep_c67786_66472676*” (G), “*wsnp_Ex_c3145_5812670*” (T), “*wsnp_Ex_c298_580660*” (A) and “*KukRi_c49927_151*” (T), and enabled a high yield over drought and optimum conditions.

## 3. Discussion

Breeding for drought tolerance is one of the top priorities in most wheat breeding programs worldwide. A number of important yield-related traits play an important role in drought resistance mechanisms and influence either directly or indirectly grain yield [22,34,35]. Therefore, the present research was carried out to identify an efficient combination of phenotypic secondary selection criteria and linked markers for drought tolerance.

### 3.1. Grain Yield and Related Traits

Phenotypic correlations showed that grain yield is positively correlated with grain number at both sites, Sidi El Aidi (semi-arid) and Taoujdate (favorable rainfed). These results are in agreement with many previous studies that highlighted the importance of grain number as a critical determinant of yield in wheat [22,23,24,25,26,27,28,29,30,31,32,33,34,35,36]. On the other hand, the correlation of yield with thousand kernel weight was very moderate. Grain weight is less plastic and more heritable than grain number [37,38]. Peltonen-Sainio et al. [39] and Sadras [40] linked the small variation in grain weight to the large number of primordia produced for grain reception compared to the number of grains actually existing. Moreover, grain weight is dependent on the limited reserves remaining at the grain filling and maturity stages. Thus, the greater the number of grains produced per unit area, the lower the availability of dry matter per grain, resulting in a reduced grain weight under stress. Therefore, yield is far more related to grain number than to grain weight [34]. Accordingly, it is mostly assumed that increases in grain number directly result in a net increase in yield potential. However, the capacity of the source to fill the potential sink is an important approach to improve yield potential further [41,42]. Breeding directly for high thousand kernel weigh is complex given the negative association between these traits. Alternative strategies have been proposed to achieve the best balance between these major yield components, namely through the selection of higher number of spikelets per spike [43], or higher grain weight at specific positions within the spikelets [44].

In addition, grain yield was positively correlated with biomass and number of fertile spikes per plant under optimum and stressed conditions. These two parameters contributed indirectly through grain number on yield improvement, especially under a drought environment. An important above ground biomass enables an active photosynthesis process and presents a positive correlation with root biomass and length to meet the nutrient requirements of the plant [45]. Additionally, maintaining a large number of fertile spikes would ensure a large number of kernels under stress. Therefore, preventing floret mortality at a pre-flowering stage can hinder significant reductions in yield [46,47].

On the other hand, our results show that grain yield expressed a strong positive correlation with ground cover, especially under the dry environment. This physiological trait was able to explain a great part of yield variation through its impact on biomass, grain number and to a lesser extent the number of fertile spikes per plant. Indeed, early development contributes to a rapid coverage of the soil surface, thereby conserving soil moisture and promoting root development. Identifying new sources of germplasm with early vigor and early ground coverage is therefore of outmost importance [48].

### 3.2. Marker–Trait Associations

Association mapping yielded a total of 73 significant MTAs (*p* < 0.001) at the Taoujdate and Sidi El Aidi stations. In line with earlier studies [49,50,51], most of these associations are positioned on the genome B and A (essentially 2B and 5B followed, respectively, by 3A and 5A), while on the D genome, the number of detected associations was very low. The higher diversity of MTAs observed in the A and B genomes could be the result of their older evolutionary background [52,53]. Moreover, the strong selection among ancestral hexaploid synthetic lines in modern breeding programs may have led to a higher proportion of rare alleles from the D genome [54].

Given its polygenic character, grain-yield-related QTLs have been reported on different chromosomal positions [18,55,56,57,58]. The identified MTAs revealed environment-specific QTLs on chromosome 4B for the rain-fed favorable site and on chromosome 7A for the semi-arid site with an average phenotypic contribution of 6.5% (*p* < 0.001). Previous studies have reported drought-resistance genes on the same chromosomes [19,58,59,60,61]. Most MTAs for grain yield showed a poor genetic stability in different environments as reported by Tadesse et al. [22] and Qassem et al. [62]. The markers “*wsnp_Ex_Rep_c67786_66472676*” (3A, 110 cM) and “*ExcalibuR_c24593_1217*” (7A, 42 cM) detected for Sidi El Aidi had a significant impact on yield improvement (+0.38 and +0.32 t/ha, respectively). The marker “*wsnp_Ex_Rep_c67786_66472676*” has been aligned to the scarecrow-like protein 1, a transcriptional regulator mastering growth repressors [63,64], whereas the marker “*ExcalibuR_c24593_1217*”, also known by the names “*BS00128708*” and “*IWB24184*”, was related to *Yr* genes and was linked to plant-drought adaptability related traits [65].

Additionally, twelve MTAs associated with fertile spikes number were identified in the present study on chromosomes 2B, 5B, 7B and 7D [55,56,61] at the rain-fed favorable site, with an average phenotypic contribution of 8% ranging from 6% “*Ra_c4397_542*” to 10% “*KukRi_Rep_c109397_59*”. On the other hand, seven MTAs were identified for the semi-arid environment on chromosomes 5B and 7B [56,57,58,59,60,61] with an average phenotypic variation of 5%. One marker on chromosome 5B at 20 cM was consistent across both the Taoujdate and Sidi El Aidi stations. This marker “*TduRuM_contig25432_1377*” is linked to *tauschii* probable polyamine transporter At1g31830 [66]. One common chromosomal position between favorable and dry site was also detected for BM on chromosome 1A at 38 cM. Additionally, the same marker identified at the Sidi El Aidi site was associated with GN and was only 3 cM far from the marker “RAC875_c34888_65” linked at the Taoujdate site.

Furthermore, significant MTAs were detected for the remaining drought-related traits namely TKW and GC. The MTAs detected were environment-specific and genetically unstable over environments. This demonstrates the presence of a very strong QTL x environment interaction for yield- and drought-related traits as reported by Edae et al. [21].

### 3.3. Co-Localization of QTLs/Genes for Yield-Related Traits

Given the polygenic character of drought tolerance expression, pleiotropic effects are particularly useful in the context of crop improvement, as they allow the breeder to select simultaneously for multiple traits [21,62]. Many studies have reported interaction effects or genetic linkages among yield-related traits [67,68,69].

In the present study, one QTL harbored two markers “*KukRi_c94792_127*” and “*wsnp_Ex_c298_580660*” on chromosome 2B (153 and 154 cM, respectively) associated with grain yield, biomass and grain number and showed a significant increase in yield performance at the sem-iarid environment (+7 and +11%) when holding, respectively, the “G” and “A” nucleotides. Furthermore, ground cover was linked to grain yield and biomass on chromosome 3A via the marker “*wsnp_Ex_Rep_c67786_66472676*”. This marker showed an increase of 0.38 t/ha on yield performance. The marker “*KukRi_c49927_151*” associated with grain yield and ground cover showed a yield improvement of 0.37 t/ha. Many studies have reported that chromosome 2B and 3A carry productivity and adaptability related genes [70,71]. These results support the significant effect of biomass, grain number, number of fertile spikes and ground cover on grain yield improvement and revealed the pleiotropy associated with the grain-yield- and drought-related traits based on their complex relationships [35].

## 4. Materials and Methods

### 4.1. Mapping Population

A panel of 197 spring bread wheat genotypes, originating from the ICARDA bread wheat breeding program, was assessed to identify closely associated markers for grain yield and various agro-physiological traits related to drought tolerance. This panel consists of synthetic derived lines, cultivars from the Central and West Asia and North Africa (CWANA) region, and elite lines from ICARDA’s wheat breeding program (Supplemental Table S3). This panel has been previously tested for heat tolerance, yield potential and quality traits and showed large genetic diversity [22].

### 4.2. Phenotyping

The mapping population was evaluated under contrasting field conditions at the Taoujdate and Sidi El Aidi experimental stations of the National Institute of Agricultural Research (INRA-Morocco). The Sidi El Aidi station is located at the semi-arid zone of Morocco (Settat region) (<300 mm), while Taoujdate station belongs to the favorable humid agro-ecological zone (Sais region) (>400 mm).

Following an alpha lattice design with two replications, the panel was planted in 6 rows plots of 3 m length and with 0.25 m spacing between rows. The trial was conducted at both experimental stations (Taoujdate and Sidi El Aidi) during two cropping seasons (2015 and 2016). The cropping seasons were dry; however, 2016 was drier than 2015 season. The Taoujdate site received 407 mm in 2015 versus 321 mm during 2016 season. On the other hand, the Sidi El Aidi site accumulated 258 and 135 mm in 2015 and 2016 cropping seasons, respectively. The average temperatures were also higher during 2016 season with 21 °C and 18 °C for Taoujdate and Sidi El Aidi, respectively, as compared to 15 and 14 °C during 2015 season.

Agronomic management was performed according to the recommended practices at each location. Chemical treatments against foliar diseases and weeds were performed as needed during the crop cycle. For each trial, days to flowering was recorded for each plot when 50% of the plants in a plot reached the Zadoks’ stage 58 (flowering stage). At harvest, aboveground biomass (BM) was determined by cutting from the ground level the plants in one linear meter from a central row of each plot. From this sample, the number of fertile spikes (NFS) and the number of grains per m² (GN) were counted. Thousand-kernel weight (TKW) was calculated by weighing 1000 grains sample. Grain yield (GY) was recorded from 4.5 m² of the harvested plot and converted to the standard unit at metric ton per hectare (t/ha). Moreover, ground cover was represented by soil cover percentage using digital camera photos and Sigma Scan Pro 5.0 software (Systat, Inc., San Jose, CA, USA, 1999).

### 4.3. Statistical Analysis

GenStat 18 (VSN International, 2016) was used to carry out ANOVA and basic statistics for yield- and drought-related traits across environments. Following the mixed-model approach for multiyear alpha-lattice designs, the genotype, year, and genotype x year were considered fixed factors, while the interactions year x replication and year x block were considered random. Additionally, IBM SPSS version 21 (IBM Corp., New York, NY, USA, 2012) was used to perform Pearson’s correlation and regression analysis.

### 4.4. Genotyping

The lines were genotyped by a service provider company (TraitGenetics Gmbh; Gatersleben) using 15K single nucleotide polymorphism (SNP) from the wheat 90 K Illumina iSelect SNP array [31]. The genotyping process, linkage disequilibrium (LD) decay rate and the population structure analysis of the investigated genotypes panel have been already described in a previous study by Tadesse et al. [22].

The SNP marker location along the chromosomes in terms of genetic distance was based on the consensus genetic map of wheat [31]. After discarding SNPs with minor allele frequency of <5% and missing values (>10%), a total of 10,568 SNPs were used for the analysis. From this set, 5350 markers with known position were randomly selected to perform the LD analysis using TASSEL V5 software [72]. The structure analysis, performed by Tadesse et al. [22], revealed the existence of three groups. Complementary analysis was performed to confirm these results using Adegenet package [73] for R software (Supplemental Figure S2). Pairwise LD was measured using the squared allele frequency correlation r² according to Weir [74]. Only *p*-values ≤ 0.001 for each pair of loci were considered significant. The LD decay was slower in the D genome (7 cM) compared to A and B genomes (3 cM) (Supplemental Figure S3).

### 4.5. Association Mapping

The GAPIT program was used to identify the best model and find marker trait associations (MTAs) for each trait. Based on Bayesian Information Content (BIC), the model with the highest value was the simplest one using only the kinship model (MLM+PCA). Best linear unbiased estimations for the genotypic factor and the corresponding marker data were used to perform GWAS analysis. All the detected MTAs at a critical *p*-value of 0.001 were considered as a significant association and the Manhattan plots were generated using GAPIT under R software (Supplemental Figure S3).

## 5. Conclusions

In light of these results, the grain number and biomass supported by the number of fertile spikes per plant and ground cover have a significant impact on yield improvement under dry conditions and constitute potential secondary selection criteria for drought resistance. The GWAS analysis identified significant associations between markers and target traits at various chromosomal positions, especially on chromosomes 2B and 5B followed by 3A and 5A, respectively. Some linkage groups involved multiple traits of interest reflecting eventual pleiotropic effects, which are of great interest for gene pyramiding. Our results indicated that the genotypes holding the positive allele of the markers “*ExcalibuR_c24593_1217*” (T), “*wsnp_Ex_Rep_c67786_66472676*” (G), “*wsnp_Ex_c298_580660*” (A), “*KukRi_c49927_151*” (T) and “*Kukri_c94792_127*” (G) were the most important ones showing significant mean yield increase varying from 7 to 11%. Further investigations should be carried out to validate the efficiency of these markers in the selection for drought tolerance under various environments and genetic backgrounds. The top high yielding lines identified from the current study will be considered for potential release targeting the semi-arid lands in Morocco after agronomic validation trials.

## Figures and Tables

**Table 1:** Pearson’s correlation among traits at Taoujdate (below diagonal) and Sidi El Aidi (above diagonal) experimental stations.

Traits †	GY	TKW	BM	GN	NFSP	GC	DTF
GY	1	0.24 **	0.88 ***	0.83 ***	0.43 ***	0.75 ***	−0.02
TKW	0.24 ***	1	0.08	−0.17 *	−0.00	0.03	−0.25 ***
BM	0.89 ***	0.11	1	0.85 ***	0.69 ***	0.54 ***	0.01
GN	0.90 ***	−0.25 ***	0.82 ***	1	0.66 ***	0.46 ***	0.15 *
NFSP	0.69 ***	−0.01	0.43 ***	0.44 ***	1	0.46 ***	0.01
GC	0.16 *	0.01	0.20 **	0.19 **	0.27 **	1	0.14 *
DTF	−0.05	−0.28 ***	0.12	0.05	−0.02	−0.19 **	1

*, **, *** Significant at the 0.05, 0.01, and 0.001 probability levels, respectively. † GY: Grain yield; TKW: thousand kernel weight; BM: biomass; GN: number of grains per m²; NFSP: number of fertile spikes per plant; GC: ground cover; DTF: days to flowering.

**Table 2:** Regression model analysis for grain yield prediction through secondary traits.

Sites	Traits †	Fpr	R²a ‡	SEE	Durbin Watson	Intercept	b §	SE	β ¶	Fpr
Sidi El Aidi	NFSP	<0.001	0.47	0.48	1.95	1.15	0.86	0.06	0.69	<0.001
	BM	<0.001	0.79	0.31	1.50	0.03	0.004	0.000	0.89	<0.001
	TKW	0.001	0.05	0.65	1.79	1.55	0.05	0.014	0.24	0.001
	GN	<0.001	0.80	0.30	1.72	0.29	0.000	0.000	0.89	<0.001
	DTF	0.49	−0.003	0.67	1.76	4.49	−0.008	0.011	−0.05	−0.49
	GC	<0.001	0.56	0.35	1.80	−5.00	0.09	0.01	0.75	<0.001
Taoujdate	NFSP	<0.001	0.18	0.45	1.60	1.83	0.70	0.10	0.43	<0.001
	BM	<0.001	0.78	0.24	1.61	−0.52	0.003	0.000	0.88	<0.001
	TKW	0.001	0.05	0.49	1.51	1.87	0.05	0.013	0.24	0.001
	GN	<0.001	0.69	0.28	1.74	0.72	0.000	0.000	0.83	<0.001
	DTF	0.80	−0.005	0.50	1.46	3.98	−0.004	0.02	−0.02	0.80
	GC	0.024	0.021	0.50	1.48	−0.08	0.04	0.02	0.16	0.02

† NFSP: number of fertile spikes per plant; BM: biomass; TKW: thousand kernel weight; GN: number of grains per m²; DTF: days to flowering; GC: ground cover; ‡ R²a: adjusted R²; SEE: Standard error estimated; b §: Standardized regression coefficient; SE: Standard error; β ¶: Non-standardized regression coefficient; Fpr: Probability of the model.

**Table 3:** Direct and indirect effects of each variable on grain yield under favorable and dry environments.

Site	Traits	Direct and Indirect Effects					Total Indirect Effects
		NFSP	BM	TKW	GN	GC	
Sidi El Aidi	NFSP	**0.005**	0.07	0.001	0.36	0.001	0.43
	BM	0.03	**0.02**	0.03	0.59	0.003	0.65
	TKW	0.000	0.003	**0.12**	0.03	0.02	0.05
	GN	0.04	0.12	0.05	**0.61**	0.001	0.21
	GC	0.001	0.001	0.03	0.001	**0.31**	0.03
Taoujdate	NFSP	**0.004**	0.04	0.001	0.14	0.002	0.18
	BM	0.006	**0.05**	0.04	0.53	0.004	0.58
	TKW	0.000	0.006	**0.17**	0.05	0.000	0.06
	GN	0.005	0.16	0.09	**0.55**	0.003	0.26
	GC	0.001	0.015	0.01	0.04	**0.000**	0.07

NFSP: number of fertile spikes per plant; BM: biomass; TKW: thousand kernel weight; GN: number of grains per m². Bold numbers on diagonals refer to direct effects, whereas the other values represent indirect effects.

**Table 4:** Regression model analysis for ground cover prediction estimation of yield and yield components.

Site	Traits †	Fpr	R²a ‡	SEE	Durbin Watson	Intercept	b §	SE	β ¶	T	Sign
Sidi El Aidi	NFSP	<0.001	0.47	0.48	1.95	1.15	0.86	0.06	0.69	13.42	<0.001
	BM	<0.001	0.79	0.31	1.50	0.03	0.004	0.000	0.89	27.32	<0.001
	TKW	0.001	0.05	0.65	1.79	1.55	0.05	0.014	0.24	3.39	0.001
	GN	<0.001	0.80	0.30	1.72	0.29	0.000	0.000	0.90	28.16	<0.001
Taoujdate	GN	0.007	0.03	1509.16	1.74	−2715.9	132.18	48.75	0.19	2.71	0.007
	NFSP	<0.001	0.07	0.296	1.93	−1.39	0.04	0.10	0.27	3.96	<0.001
	TKW	0.902	−0.005	2.72	2.00	33.66	0.01	0.09	0.009	0.12	0.902
	BM	0.004	0.04	127.74	1.67	5.39	12.01	4.13	0.203	2.912	0.004

† NFSP: number of fertile spikes per plant; BM: biomass; TKW: thousand kernel weight; GN: number of grains per m²; ‡ R²a: adjusted R²; SEE: Standard error estimated; b §: Standardized regression coefficient; SE: Standard error; β ¶: Non-standardized regression coefficient; Fpr: Probability of the model.

## Supplementary Materials

The following supporting information can be downloaded at: https://www.mdpi.com/article/10.3390/plants11070986/s1. Figure S1: Manhattan plots showing SNP markers associated with efficient secondary traits (A,A’) grain number; (B,B’) Biomass; (C,C’) number of fertile spikes per plant and (D,D’) ground cover at the Sidi El Aidi and Taoujdate stations, respectively; Figure S2: Population structure (A) plot of the Bayesian information criterion (BIC) for each population number from 1 to 10 using a *K* means cluster, (B) biplot of the first two principal components (PCs) of the genotypic data, (C) population density, and (D) the assignment of each individual to the corresponding subpopulation; Figure S3: Linkage desequilibrium (r²) plot in 197 wheat genotypes across (A) genome A, (B) genome B and (C) genome D; Table S1: Summary of main MTAs identified at *p* < 0.001 for the average grain yield- and drought-related traits in Taoujdate environment during 2015 and 2016 seasons; Table S2: Summary of main MTAs identified at *p* < 0.001 for the average grain yield- and drought-related traits at the Sidi El Aidi environment during 2015 and 2016 seasons; Table S3: List of the genetic panel and lines pedigrees.
