# Supplementary material for: Drought-Tolerance QTLs Associated with Grain Yield and Related Traits in Spring Bread Wheat
Source: Plants (Basel). 2022 Apr 4;11(7):986. doi: 10.3390/plants11070986 (PMC9002858; doi:10.3390/plants11070986)
Supplement: Supplementary file 1 [file plants-11-00986-s001.zip › plants-1580659-supplementary.pdf]

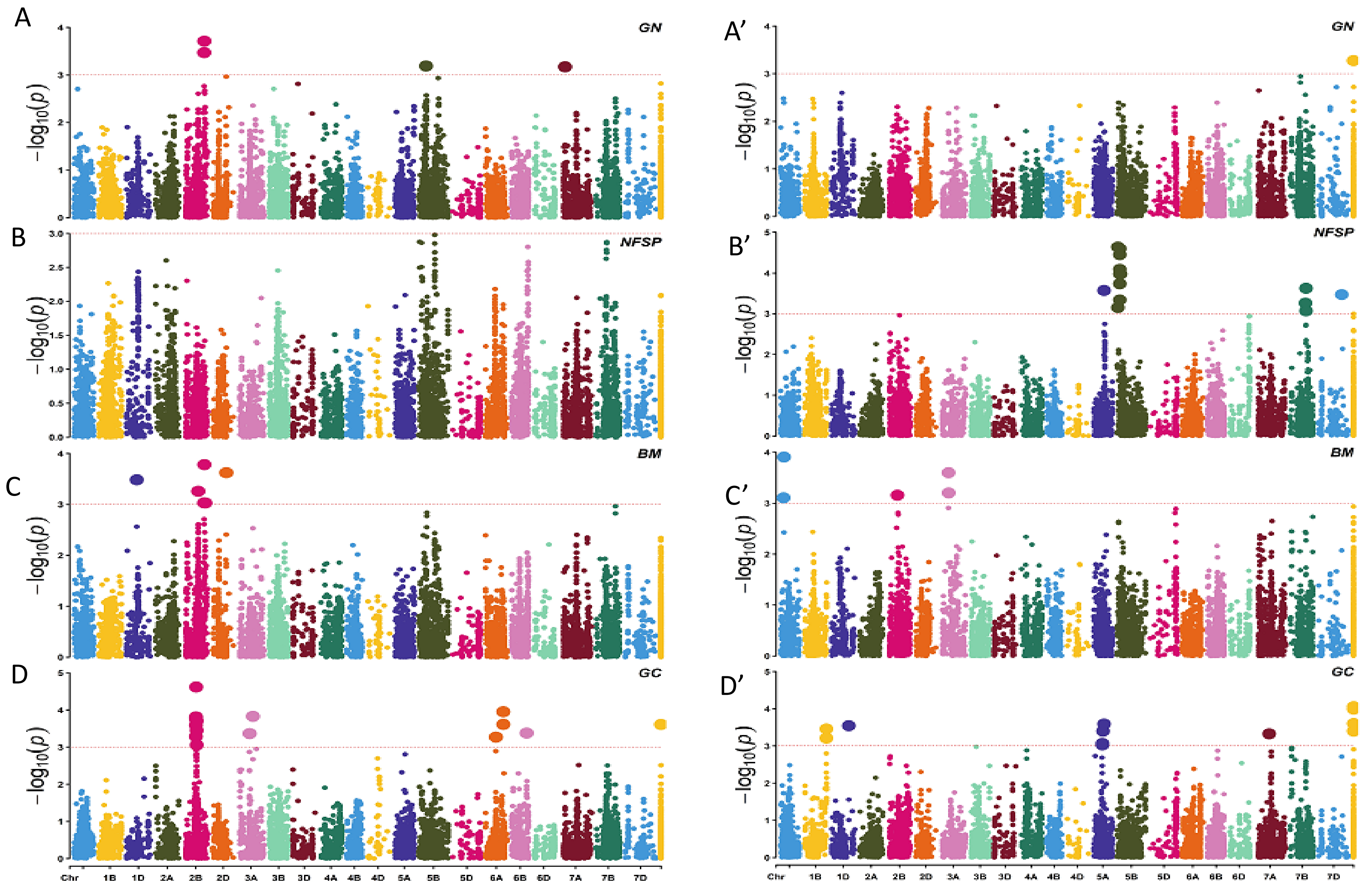

Supplementary Figure S1: Manhattan plots showing SNP markers associated with efficient secondary traits A, A’) grain number ; B, B’) Biomass ; C, C’) number of fertile spikes per plant and D, D’) ground cover at Sidi El Aidi and Taoujdate Stations respectively

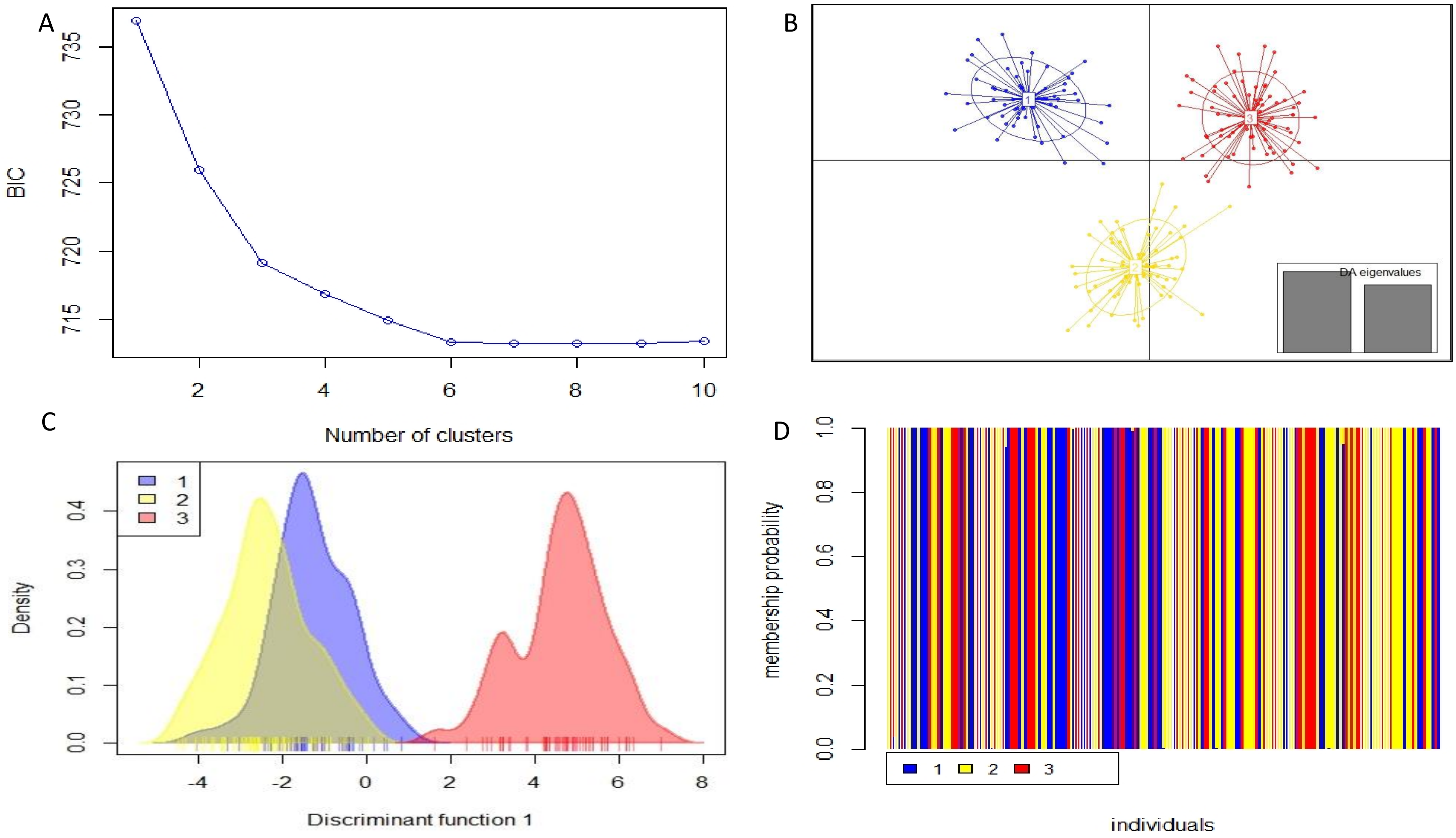

Supplementary Figure S2: Population structure (A) plot of the Bayesian information criterion (BIC) for each population number from 1 to 10 using a *K* means cluster, (B) biplot of the first two principal components (PCs) of the genotypic data, (C) population density, and (D) the assignment of each individual to the corresponding subpopulation

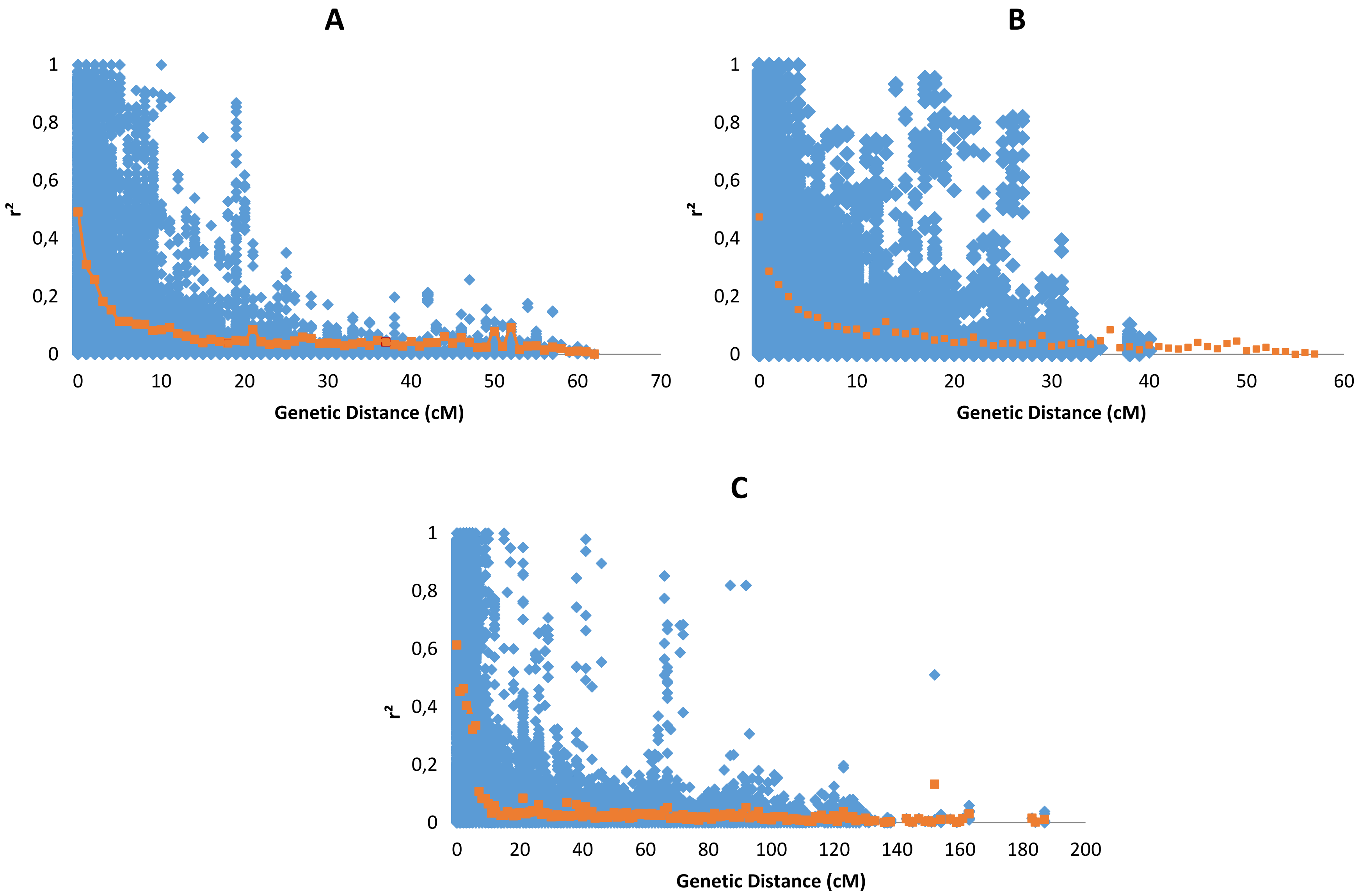

Supplementary Figure S3: Linkage desequilibrium(r^2) plot in 197 wheat genotypes across(A) genome A, (B) genome B and (C) genome D

Supplemental Table S1: Summary of main MTAs identified at *p*<0.001 for the average grain yield and drought related traits at Sidi El Aidi environment during 2015 and 2016 seasons

Trait†	Marker	Chr.	Pos. cM	*p*	Marker R^2 %	Allele	Effect	Frequency %	Alternative Allele	Frequency %
GY	wsnp_BG263521B_Ta_2_1	2B	97	5.81E-04	0.06	C	-3.92E4	45.4	T	54.6
	ExcalibuR_c24593_1217	7A	42	7.04E-04	0.06	C	1.47	44.6	T	55.4
BM	GENE-0675_104	2B	108	5.52E-04	0.06	C	33.34	9.7	T	90.3
	RFL_Contig5495_563	2B	161	9.39E-04	0.06	A	991.89	55.0	G	44.9
GN	ExcalibuR_c24593_1217	7A	42	6.76E-04	0.06	C	39385.42	44.6	T	55.4
	ExcalibuR_c32630_104	5B	52	6.45E-04	0.06	A	3.33	23.6	G	76.4
	KukRi_c94792_127	2B	153	3.40E-04	0.07	A	1.99	46.2	G	53.9
	wsnp_Ex_c298_580660	2B	154	1.95E-04	0.07	A	1.96	47.7	G	52.3
TKW	RFL_Contig2736_827	1B	96	9.02E-04	0.06	C	-1.01E0	78.1	T	21.9
GC	BS00022417_51	2B	91	1.73E-04	0.08	A	1.55	7.7	G	92.3
	BS00022949_51	2B	91	1.53E-04	0.08	C	-9.83E3	92.4	T	7.6
	BS00099097_51	2B	91	1.53E-04	0.08	C	9827.48	7.6	T	92.4
	IAAV2784	2B	91	1.53E-04	0.08	A	9827.48	7.6	G	92.4
	KukRi_c40953_658	2B	91	1.57E-04	0.08	C	-1.41E4	92.4	T	7.7
	KukRi_c67546_279	2B	91	2.58E-04	0.07	C	0.80	91.7	T	8.3
	KukRi_c67546_342	2B	91	1.53E-04	0.08	A	-2.05E0	7.6	G	92.4
	KukRi_c7139_6288	2B	91	1.57E-04	0.08	A	1.98	92.2	G	7.8

Supplemental Table S1 (continued) : Summary of main MTAs identified at $p<0.001$ for the average grain yield and drought related traits at Sidi El Aidi environment during 2015 and 2016 seasons

Trait†	Marker	Chr.	Pos.	*p*	Marker R^2	Allele	Effect	Frequency	Alternative Allele	Frequency
			cM		%			%		%
	RAC875_c15649_1101	2B	91	1.53E-04	0.08	C	1.63	7.6	T	92.4
	RAC875_c31252_173	2B	91	1.71E-04	0.08	G	8628.15	7.7	A	92.3
	RFL_Contig1385_326	2B	91	1.53E-04	0.08	A	2.05	92.4	G	7.6
	BS00065418_51	2B	92	1.53E-04	0.08	G	-2.05E0	7.6	A	92.4
	GENE-4359_102	2B	92	1.57E-04	0.08	A	1.60	7.7	G	92.4
	RAC875_Rep_c118376_304	2B	92	1.53E-04	0.08	A	-1.63E0	92.4	G	7.6
	wsnp_CAP11_Rep_c8700_3756682	2B	92	1.53E-04	0.08	C	-1.63E0	92.4	T	7.6
**GG**	Ku_c34010_1016	2B	93	2.41E-05	0.10	A	2.35	6.6	G	93.4
	BS00064851_51	2B	97	1.94E-04	0.07	A	0.54	10.7	G	89.3
	Ku_c4777_2494	2B	97	1.99E-04	0.07	C	-5.33E-1	89.3	T	10.7
	wsnp_Ex_Rep_c67786_66472676	3A	110	1.48E-04	0.08	A	0.68	10.7	G	89.3
	KukRi_c41482_311	6A	134	1.12E-04	0.08	A	-2.40E-1	9.8	G	90.2
	wsnp_JD_Rep_c65886_41872083	6A	134	2.44E-04	0.07	C	35390.06	10.2	T	89.9
	wsnp_Ra_c21546_30949373	NP	1	2.46E-04	0.07	A	45456.10	10.8	G	89.2

† GY Grain yield; BM biomass; NFSP number of fertile spikes per plant; GN Number of grains per m², TKW Thousand kernel weight; GC Ground cover; NP: Unknown position

Supplemental Table S2: Summary of main MTAs identified at p<0.001 for the average grain yield and drought related traits in Taoujdate environment during 2015 and 2016 seasons

Trait†	Marker	Chr.	Pos. cM	p	Marker R^2 %	Allele	Effect	Frequency %	Alternative Allele	Frequency %
**GY**	wsnp_Ex_c3145_5812670	1A	96	7.43E-04	0.06	C	-3.09E0	90.0	T	10.0
	BS00067775_51	4B	6	3.03E-04	0.07	C	-5.49E-1	78.0	T	22.0
	wsnp_Ra_c2078_4037878	4B	6	5.57E-04	0.06	C	-4.04E-1	77.5	T	22.5
	RAC875_c78248_154	NP	1	1.81E-04	0.08	A	0.63	20.4	G	79.6
	TduRuM_contig14482_1013	NP	2	4.47E-04	0.07	C	1.34	67.3	T	32.7
**BM**	RAC875_c34888_65	1A	35	7.82E-04	0.06	A	7.41	5.1	G	94.9
	tplb0043h23_1346	1A	38	1.24E-04	0.08	C	10.67	8.7	T	91.3
	ExcalibuR_c20196_503	2B	69	0.00304	0.05	A	5.79	92.9	G	7.1
	IACX1098	2B	75	6.97E-04	0.06	A	-2.08E0	46.4	G	53.6
	wsnp_Ex_Rep_c70571_6948 8416	2B	81	0.00154	0.05	A	-2.06E-1	26.6	G	73.4
	BS00010055_51	2B	82	0.00168	0.05	A	1.59	12.8	G	87.2
	BS00048757_51	3A	60	2.51E-04	0.07	A	1.09	48.9	G	51.1
	BS00065956_51	3A	61	6.24E-04	0.06	A	-1.98E-1	47.4	G	52.6
**NFSP**	Ra_c22700_818	5A	83	2.68E-04	0.07	A	41189.75	27.8	G	72.2
	KukRi_Rep_c109397_59	5B	6	2.30E-05	0.10	G	43272.53	21.6	A	78.4
	wsnp_Ku_c64203_64579087	5B	6	7.11E-04	0.06	C	31166.58	19.0	T	81.0
	BobWhite_Rep_c50066_63	5B	20	1.05E-04	0.08	A	32187.29	11.7	G	88.3
	ExcalibuR_c3948_1315	5B	20	3.54E-05	0.09	C	-3.69E4	77.6	T	22.4
	KukRi_c2514_490	5B	20	8.11E-05	0.09	A	-3.71E4	76.9	G	23.1
	TduRuM_contig25432_1218	5B	20	2.52E-05	0.10	C	39371.81	21.5	T	78.5
	TduRuM_contig25432_1377	5B	20	1.83E-04	0.08	A	-3.41E4	75.8	G	24.2
	wsnp_BF201102B_Ta_2_5	5B	20	4.58E-04	0.07	A	-3.32E4	87.7	G	12.3
	wsnp_BE499835B_Ta_2_5	5B	25	1.05E-04	0.08	G	1.55	11.7	A	88.3

Supplemental Table S2 (continued) : Summary of main MTAs identified at *p*<0.001 for the average grain yield and drought related traits in Taoujdate environment during 2015 and 2016 seasons

Trait†	Marker	Chr.	Pos. cM	*p*	Marker R^2 %	Allele	Effect	Frequency %	Alternative Allele	Frequency %
**NFSP**	TduRuM_contig5360_329	7B	114	5.53E-04	0.07	A	-5.58E4	70.4	G	29.6
	BS00085556_51	7B	119	2.37E-04	0.08	G	54180.92	26.3	A	73.7
	RAC875_c89312_61	7B	119	8.55E-04	0.07	C	55713.65	25.1	T	74.9
	IAAV6659	7D	180	3.39E-04	0.08	A	50735.16	25.9	G	74.1
**GN**	CAP12_c3807_144	NP	1	5.32E-04	0.06	C	5.02	51.8	T	48.2
**TKW**	CAP8_c1799_237	3B	62	5.69E-04	0.06	A	-4.99E-1	5.1	G	94.9
**GC**	TduRuM_contig9144_222	1B	171	3.49E-04	0.07	G	13.28	53.9	A	46.1
	wsnp_BE446672B_Ta_2_1	1B	171	6.15E-04	0.06	C	-3.32E4	43.5	T	56.5
	BS00022188_51	1D	132	2.86E-04	0.07	C	-3.47E-1	93.7	T	6.3
	GENE-3318_556	5A	70	9.37E-04	0.06	C	-2.01E0	9.1	T	90.9
	KukRi_c14889_1086	5A	70	8.54E-04	0.06	C	18.53	9.2	T	90.8
	KukRi_c14889_116	5A	70	9.00E-04	0.06	C	-1.06E4	89.9	T	10.1
	ExcalibuR_c9210_168	5A	75	4.00E-04	0.06	A	3.18	90.4	G	9.6
	Ra_c22700_818	5A	83	2.58E-04	0.07	A	0.63	27.8	G	72.2
	KukRi_c57674_324	7A	113	4.70E-04	0.06	C	0.37	85.1	T	14.9
	BS00072156_51	NP	2	8.74E-05	0.08	A	-2.33E1	92.4	G	7.6
	BS00083514_51	NP	2	2.49E-04	0.07	C	-2.38E1	93.9	T	6.1
	ExcalibuR_Rep_c68708_80	NP	2	4.00E-04	0.06	A	9302.79	90.4	G	9.6
	KukRi_c41594_74	NP	2	3.95E-04	0.06	C	9643.83	90.3	T	9.7

† GY Grain yield; BM biomass; NFSP number of fertile spikes per plant; GN Number of grains per m², TKW Thousand kernel weight; GC Ground cover; NP: Unknown position

Supplemental Table S3 : List of the genetic panel and lines pedigrees

Genotype	Name/Pedigree	Genotype	Name/Pedigree
1	ATTILA*2/PBW65//PFAU/MILAN	41	KAUZ//ALTAR 84/AOS/3/MILAN/DUCULA
2	SERI.1B//KAUZ/HEVO/3/AMAD/4/PFAU/MILAN	42	QAFZAH-16/ICARDA-SRRL-5
3	QAFZAH-21/ICARDA-SRRL-9	43	ESDA/SHWA//BCN/3/MILAN/PASTOR
4	KAUZ'S'/SERI/3/KAUZ//KAUZ/STAR	44	KAUZ//ALTAR 84/AOS/3/MILAN/DUCULA
5	SERI.1B//KAUZ/HEVO/3/AMAD/4/PFAU/MILAN	45	SERI.1B*2/3/KAUZ*2/BOW//KAUZ/4/ANGI-2
6	ATTILA*2/PBW65//PFAU/MILAN	46	HUBARA-5/PASTOR-2
7	CROC1/AE.SQUARROSSA (205)//KAUZ/3/ATTILA/4/FLAG-1	47	GIRWILL-13/2*PASTOR-2
8	BACANORA T 88/SHIHAB-8	48	HUBARA-7/4/PASTOR/3/KAUZ*2/OPATA//KAUZ
9	KAUZ'S'/BOCRO-3//ANGI-2	49	HIDDAB/CHAM-8
10	GOUBARA-1/ANGI-1	50	QIMMA-12
11	ATTILA 50Y//ATTILA/BCN/3/PFAU/MILAN	51	ANGI-2/HUBARA-3
12	HUBARA-5/ANGI-1	52	ESDA/SHWA//BCN/3/MILAN/PASTOR
13	SERI.1B//KAUZ/HEVO/3/AMAD/4/KAUZ/FLORKWA-1	53	SOMAMA-9/ICARDA-SRRL-2
14	KAUZ'S'/SERI/3/KAUZ//KAUZ/STAR	54	SERI.1B//KAUZ/HEVO/3/AMAD/4/PFAU/MILAN
15	GIRWILL-13/2*PASTOR-2	55	ATTILA//VEE#5/DOBUC'S'/3/QADANFER-9
16	CHILERO-1/4/VEE'S'/3/HORK/4MH//KAL-BB/5/PFAU/MILAN	56	HIDDAB/CHAM-8
17	HIDDAB/CHAM-8	57	DEBEIRA/ANGI-2
18	CROC1/AE.SQUARROSSA (205)//KAUZ/3/ATTILA/4/FLAG-1	58	QAFZAH-2/FERROUG-2//ZEMAMRA-8
19	SERI 82/SHUHA'S'//GRU90-204782/4/PASTOR/3/KAUZ*2/OPATA//KAUZ	59	KAUZ//MON/CROW'S/3/VEE/PJN//2*KAUZ
20	ZEMAMRA-1/2*SOMAMA-3	60	KAUZ'S'/SERI/3/KAUZ//KAUZ/STAR
21	ZOLOTARA//SHA3/SERI/3/SKAUZ/2*STAR	61	ANGI-5/ZEMAMRA-8
22	ATTILA//VEE#5/DOBUC'S'/3/QADANFER-9	62	KAUZ'S'/FLORKWA-1//GOUMRIA-3
23	ATTILA//VEE#5/DOBUC'S'/3/QADANFER-9	63	YMI #6/GEN//TIA.1/3/VEE#5//DOVE/BUC/4/MILAN/PASTOR
24	ESDA/SHWA//BCN/3/MILAN/PASTOR	64	SERI 82/SHUHA'S'//GRU90-204782/3/MUNIA/CHTO//MILAN
25	PASTOR-2	65	SERI.1B*2/3/KAUZ*2/BOW//KAUZ/4/ANGI-1
26	HUBARA-13/4/TRAP#1/BOW//PFAU/3/MILAN	66	HUBARA-5/PASTOR-2
27	HUBARA-5/5/CHEN/AEGILOPS SQUARROSA (TAUS)//BCN/3/VEE#7/BOW/4/PASTOR	67	HUBARA-15/CATBIRD//PASTOR-2
28	IZAZ-1/KATILA-11//GOUMRIA-3	68	QAFZAH-23/SOMAMA-3//GOUMRIA-3
29	SERI.1B//KAUZ/HEVO/3/AMAD/4/PFAU/MILAN	69	ATTILA*2/PBW65//PFAU/MILAN
30	BOUSHODA-1/5/CHEN/AEGILOPS SQUARROSA (TAUS)//BCN/3/VEE#7/BOW/4/PASTOR	70	PASTOR-2/HUBARA-5
31	ANGI-2/HUBARA-3	71	SERI.1B*2/3/KAUZ*2/BOW//KAUZ/4/KAUZ/FLORKWA-1
32	QIMMA-12/PASTOR-6//QIMMA-12	72	KAUZ'S'/FLORKWA-1//GOUMRIA-3
33	SOMAMA-9/ICARDA-SRRL-2	73	SERI.1B//KAUZ/HEVO/3/AMAD/4/PFAU/MILAN
34	VEE/PJN//2*KAUZ/3/SHUHA-4/FOW-2	74	HAAMA-17/ANGI-2
35	QAFZAH-2/FERROUG-2//ZEMAMRA-8	75	ATTILA-7
36	SOMAMA-9/NEJMAH-18	76	SERI.1B*2/3/KAUZ*2/BOW//KAUZ/4/ANGI-2
37	KAUZ'S'/SERI/3/KAUZ//KAUZ/STAR	77	ATTILA*2/PBW65//PFAU/MILAN
38	HUBARA-5/5/CHEN/AEGILOPS SQUARROSA (TAUS)//BCN/3/VEE#7/BOW/4/PASTOR	78	ATTILA//VEE#5/DOBUC'S'/3/QADANFER-9
39	SERI.1B//KAUZ/HEVO/3/AMAD/4/PFAU/MILAN	79	KAUZ//MON/CROW'S'/3/KAUZ//KAUZ/STAR/5/SHAMIEKH-7
40	QAFZAH-35/AMIR-2	80	SERI.1B*2/3/KAUZ*2/BOW//KAUZ/4/ANGI-1

Supplemental Table S3 (continued): List of genetic material and pedigrees

Genotype	Name/Pedigree	Genotype	Name/Pedigree
81	SERI.1B//KAUZ/HEVO/3/AMAD/4/PFAU/MILAN	121	P1.861/RDWG//PBW343/3/MUNIA/ALTAR 84//AMSEL
82	GOUMRIA-15/ANGI-2	122	OPATA/RAYON//KAUZ/3/2*MILAN/DUCULA
83	HUBARA-16/4/PASTOR/3/KAUZ*2/OPATA//KAUZ	123	KAUZ'S'/SHUHA-15
84	SERI 82/SHUHA'S'//GRU90-204782/3/MUNIA/CHTO//MILAN	124	VEE/PJN//2*TUI/3/WH576/4/AL-ZEHRAA-5
85	HIDDAB/CHAM-8	125	Sids-1 (CHECK-5)
86	SOMAMA-9/ICARDA-SRRL-2	126	ANGI-2
87	KAUZ'S'/SERI/3/KAUZ//KAUZ/STAR	127	SERI.1B*2/3/KAUZ*2/BOW//KAUZ/4/ANGI-1/5/KABOWSH-1
88	VEE/PJN//2*KAUZ/3/SHUHA-4/FOW-2	128	MILAN/DUCULA//AL-ZEHRAA-1
89	VEE/PJN//2*KAUZ/3/SHUHA-4/FOW-2	129	BOW/PRL//BUC/3/WH576
90	ATTILA*2/PBW65//PFAU/MILAN	130	CHEN/AEGILOPS SQUARROSA (TAUS)//FCT/3/2*WEAVER/4/IPA-95
91	SERI 82/SHUHA'S'//GRU90-204782/4/PASTOR/3/KAUZ*2/OPATA//KAUZ	131	QADANFER-11
92	ATTILA//VEE#5/DOBUC'S'/3/QADANFER-9	132	QADANFER-11/REBWAH-11
93	QAFZAH-33*2/SALSAL-2	133	PASTOR/3/KAUZ*2/OPATA//KAUZ
94	ATTILA//VEE#5/DOBUC'S'/3/QADANFER-9	134	P1.861/RDWG/3/VEE/PJN//2*KAUZ/4/CMH82A.1294/2*KAUZ//MUNIA/CHTO/3/MILAN
95	SERI.1B*2/3/KAUZ*2/BOW//KAUZ/4/ANGI-1	135	VEE7/KAUZ/3/KAUZ//MON/CROW'S'/4/QAFZAH-33
96	BOUSHODA-1/5/CHEN/AEGILOPS SQUARROSA (TAUS)//BCN/3/VEE#7/BOW/4/PASTOR	136	MILAN//PSN/BOW
97	PASTOR-2/HUBARA-5	137	KAUZ//MON/CROW'S'/3/SOMAMA-3/4/MILAN/DUCULA
98	SERI 82/SHUHA'S'//GRU90-204782/3/MUNIA/CHTO//MILAN	138	GOUBARA-1/ANGI-1//QAFZAH-21
99	SERI.1B*2/3/KAUZ*2/BOW//KAUZ/4/ANGI-2	139	ATTILA 50Y//ATTILA/BCN/3/KAUZ//MON/CROW'S'/4/MILAN/PASTOR
100	KABOWSH-1 (CHECK-4)	140	VEE7/KAUZ//PFAU/MILAN/3/MILAN/PASTOR
101	QAFZAH-35/AMIR-2	141	VEE7/KAUZ//PFAU/MILAN/3/MILAN/PASTOR
102	LAKTA-1/QAFZAH-21	142	QADANFER-11/REBWAH-11
103	SOMAMA-9	143	P1.861/RDWG/3/VEE/PJN//2*KAUZ/4/CMH82A.1294/2*KAUZ//MUNIA/CHTO/3/MILAN
104	SERI.1B//KAUZ/HEVO/3/AMAD/4/ATTILA//PSN/BOW/3/ATTILA/5/KAUZ'S'/SHUHA-15	144	SERI.1B*2/3/KAUZ*2/BOW//KAUZ/4/ANGI-1/5/KABOWSH-1
105	VEE7/KAUZ//PFAU/MILAN/3/MILAN/PASTOR	145	ZEMAMRA-1
106	MUNIA/ALTAR 84//AMSEL	146	VEE/PJN//2*TUI/3/WH576
107	ATTILA//VEE#5/DOBUC'S'/3/PYN/BAU//MILAN/4/ZEMAMRA-8	147	CHEN/AEGILOPS SQUARROSA (TAUS)//FCT/3/2*WEAVER
108	ATTILA-7//MILAN/PASTOR/3/HXL8088/DUCULA	148	P1.861/RDWG//DAJAJ-10/3/MILAN/PASTOR
109	VEE7/KAUZ//PFAU/MILAN/3/MILAN/PASTOR	149	PASTOR-6
110	URES/BOW//OPATA/3/HD2206/HORK'S'	150	Debira
111	SERI.1B//KAUZ/HEVO/3/AMAD/4/ATTILA//PSN/BOW/3/ATTILA/5/KAUZ'S'/SHUHA-15	151	QAMAR-6
112	SHIHAB-16	152	KAUZ//ALTAR 84/AOS/3/TNMU/MILAN/4/MILAN//PSN/BOW
113	VEE7/KAUZ//PFAU/MILAN/3/MILAN/PASTOR	153	PFAU/MILAN
114	KAUZ'S'/SERI/3/KAUZ//KAUZ/STAR	154	KAUZ//ALTAR 84/AOS/3/TNMU/MILAN/4/MILAN//PSN/BOW
115	MILAN/DUCULA	155	KASYON/GENARO 81//TEVEE-1/../3/2*QADANFER-11
116	MILAN/DUCULA//AL-ZEHRAA-1	156	VEE/PJN//2*KAUZ
117	QADANFER-11/REBWAH-11	157	SERI.1B//KAUZ/HEVO/3/AMAD/4/ATTILA//PSN/BOW/3/ATTILA/5/KAUZ'S'/SHUHA-15
118	SERI.1B//KAUZ/HEVO/3/AMAD/4/ATTILA//PSN/BOW/3/ATTILA/5/KAUZ'S'/SHUHA-15	158	OPATA/RAYON//KAUZ/3/CHAM-6/FLORKWA-2/5/SHAMIEKH-7
119	P1.861/RDWG//PBW343/3/MUNIA/ALTAR 84//AMSEL	159	P1.861/RDWG//PBW343/3/MUNIA/ALTAR 84//AMSEL
120	KAUZ//ALTAR 84/AOS 3/KAUZ/3/SHUHA-4//NS732/HER/4/QAFZAH-33	160	WEAVER/WL 3928//SW 89.3064/3/SOMAMA-3/4/BOW/PRL//BUC/3/WH576

Supplemental Table S3 (continued): List of genetic material and pedigrees

Genotype	Name/Pedigree
161	FLAG-1
162	KAUZ'S'/FLORKWA-1
163	QAFZAH-35
164	ZEMAMRA-8
165	ATTILA//VEE#5/DOBUC'S'/3/PYN/BAU//MILAN/4/ZEMAMRA-8
166	QAFZAH-33
167	KAUZ//ALTAR 84/AOS 3/KAUZ/3/ATTILA 50Y//ATTILA/BCN/4/PASTOR-6
168	VEE/PJN//2*TUI/3/WH576/4/AL-ZEHRAA-5
169	P1.861/RDWG//DAJAJ-10/3/MILAN/PASTOR
170	ATTILA 50Y//ATTILA/BCN/3/KAUZ//MON/CROW'S'/4/MILAN/PASTOR
171	OPATA/RAYON//KAUZ/3/2*MILAN/DUCULA
172	QADANFER-11/REBWAH-11
173	SHAMISS-3
174	SERI.1B//KAUZ/HEVO/3/AMAD/4/ATTILA//PSN/BOW/3/ATTILA/5/KAUZ'S'/SHUHA-15
175	GOUMRIA-3
176	OPATA/RAYON//KAUZ/3/2*MILAN/DUCULA
177	QADANFER-11/REBWAH-11
178	KATILA-8/4/SKAUZ/BAV92/3/CROC-1/AE.SQUARROSA(224)//OPATA/5/MUNIA/ALTAR 84//MILAN
179	FAYEQ-1
180	RABIH-7
181	ATTILA-7//MILAN/PASTOR/3/ICARDA-SRRL-2
182	QADANFER-11/REBWAH-11
183	QADANFER-9
184	KASYON/GENARO 81//TEVEE-1/../3/2*QADANFER-11
185	KAUZ//ALTAR 84/AOS 3/KAUZ/3/SHUHA-4//NS732/HER/4/QAFZAH-33
186	SERI.1B*2/3/KAUZ*2/BOW//KAUZ/4/KAUZ/GYS//KAUZ/5/MUNIA/ALTAR 84//MILAN
187	VEE/PJN//2*TUI/3/WH576/4/AL-ZEHRAA-5
188	HAAMA-17/QIMMA-12
189	KAUZ//MON/CROW'S'/3/SHUHA-4//NS732/HER/4/MILAN/PASTOR
190	QADANFER-11/REBWAH-11
191	SERI.1B//KAUZ/HEVO/3/AMAD/4/ATTILA//PSN/BOW/3/ATTILA/5/KAUZ'S'/SHUHA-15
192	KAUZ'S'/SERI//STAR'S'/FLORKWA-2/3/FLAG-1
193	MILAN/PASTOR
194	ANGI-1
195	SAMIRA-9
196	MILAN/DUCULA//AL-ZEHRAA-1
197	KAUZ//MON/CROW'S'/3/KAUZ//KAUZ/STAR/5/SHAMIEKH-7
198	SHIHAB-19
199	VEE7/KAUZ/3/KAUZ//MON/CROW'S'/4/QAFZAH-33
200	HIDDAB
